# Patients with Hepatitis C Undergoing Direct-Acting Antiviral Treatment Have a Lower SARS-CoV-2 Infection Rate

**DOI:** 10.3390/life13122326

**Published:** 2023-12-11

**Authors:** Chin-Wen Hsu, Wan-Wen Yang, Chia-Yi Hou, I-Jung Feng, Ting-Yi Huang, Pei-Lun Lee, How-Ran Guo, Chien-Yuan Huang, Shih-Bin Su

**Affiliations:** 1 Department of Family Medicine, Chi-Mei Medical Center, Liouying, Tainan 736402, Taiwan; 2 Department of Clinical Pathology, Chi-Mei Medical Center, Liouying, Tainan 736402, Taiwan; 3 Institute of Precision Medicine, National Sun Yat-Sen University, Kaohsiung 804201, Taiwan; 4 Department of Hepato-Gastroenterology, Chi-Mei Medical Center, Liouying, Tainan 736402, Taiwan; 5 Department of Environmental and Occupational Health, College of Medicine, National Cheng Kung University, Tainan 70428, Taiwan; hrguo@ncku.edu.tw; 6 Division of Occupational Medicine, Chi-Mei Medical Center, Liouying, Tainan 736402, Taiwan

**Keywords:** COVID-19, ACE 2 receptors, hepatitis C virus, direct-acting antivirals, sofosbuvir, SARS-CoV-2

## Abstract

This study retrospectively analyzed the medical records of 602 patients with first-time positive results for the HCV nucleic acid test between 1 May 2021 and 31 March 2023, exploring the association between DAA treatment and SARS-CoV-2 infection. The results showed that 9.8% of HCV patients were co-infected with SARS-CoV-2. Gender, age, vaccination status, and HCV genotype did not significantly affect SARS-CoV-2 infection. However, patients undergoing DAA treatment showed significantly lower rates of SARS-CoV-2 infection and mortality compared to those not undergoing DAA treatment. The analysis also compared patients undergoing different DAA treatments, with Epclusa and Maviret showing superior protection against SARS-CoV-2. Furthermore, this study explored the severity and mortality of SARS-CoV-2 infection in patients undergoing and having completed DAA treatment. It revealed that patients diagnosed with COVID-19 during DAA treatment experienced only mild symptoms, and none died, suggesting a potential protective effect of DAA treatment against severe outcomes of SARS-CoV-2 infection. The findings contribute to the understanding of the interplay between HCV, DAA treatment, and SARS-CoV-2 infection, highlighting the need for continued monitoring and healthcare measures for individuals with chronic conditions during the ongoing COVID-19 pandemic.

## 1. Introduction

Since the outbreak of the coronavirus disease 2019 (COVID-19) pandemic in late 2019, nations around the world have been developing vaccines and drugs to control its spread and reduce the severity, hospitalization rate, and mortality of infected patients [1,2]. While the pandemic is still ongoing, the severity of the disease has decreased, since most individuals have gained immunity through infection or immunization. While most infected individuals exhibited mild or no symptoms, some developed severe pneumonia, acute respiratory distress syndrome, multiple organ failure, or shock, sometimes resulting in death [3,4].

While individuals of all ages are at risk of becoming critically ill after infection with severe acute respiratory syndrome coronavirus 2 (SARS-CoV-2), this risk increases with age in adults [5]. The United States Centers for Disease Control and Prevention (CDC) have shown that individuals with comorbidities such as cancer, cerebrovascular disease, chronic kidney disease (CKD), chronic lung disease, chronic liver disease, diabetes, heart disease, acquired immunodeficiency syndrome (AIDS), dementia, organ transplantation, and asthma are at higher risk of becoming critically ill. Chronic liver disease includes liver cirrhosis, non-alcoholic fatty liver disease, alcoholic liver disease, and autoimmune hepatitis. However, there is no consensus on the association between becoming critically ill after SARS-CoV-2 infection and being diagnosed with hepatitis B and C [6]. Another study found that patients with chronic liver disease have increased mortality and disease severity from COVID-19. Severe COVID-19 often deregulates the liver function of individuals with cirrhosis or chronic liver failure and even reactivates hepatitis in individuals infected with hepatitis B virus or hepatitis C virus (HCV) [7]. The spike protein on the surface of SARS-CoV-2 binds with the angiotensin-converting enzyme 2 (ACE2) receptors and enters the host cell. These receptors are expressed on cells in the alveolar epithelium, gastrointestinal tract, cardiac muscles, liver, kidney, and other tissues [4,5,6]. 

In 2015, the prevalence of HCV infection worldwide was 1%, and 71 million individuals were diagnosed with chronic HCV infections [8]. When the COVID-19 pandemic began in 2019, there was an increase in the mortality rate of patients infected with HCV, which may be due to co-infection when SARS-CoV-2 enters a hepatocyte through the ACE2 receptors [9]. Furthermore, neuropilin 1 (NRP1) is expressed in various tissues. Benedicto et al. suggested that NRP1 is associated with liver fibrosis and liver cancer pathogenesis [10]. Davies et al. noted that SARS-CoV-2 infects cells through NRP1, which indicates that NRP1 and HCV are associated with SARS-CoV-2 infection to some extent [11]. Hepatitis C is highly prevalent in Taiwan, with a higher prevalence (18.3%) of HCV genotype 6 in Southern Taiwan [12]. There is currently no conclusion on the genotype differences in patients co-infected with hepatitis C and SARS-CoV-2. A 2022 study proposed that HCV and SARS-CoV-2 replication needs to be facilitated by RNA-dependent RNA polymerase (RdRp). Direct-acting antivirals (DAAs) such as sofosbuvir/velpatasvir (SOF/VEL) preliminarily reduce mild-to-moderate COVID-19 infection [13]. While the evidence suggests that DAAs may serve as an early suppressant of SARS-CoV-2 infection, the actual mechanisms and outcomes remain unknown.

The WHO aims to eliminate hepatitis C by 2030. Chien et al. (2021) reported that the incidence of HCV infection in Taiwan is 3.3%, and the country has amplified its HCV screening and prevention measures [14]. In addition to the government’s measures to minimize the healthcare strain in response to the COVID-19 pandemic, patients infected with HCV were unwilling to complete their follow-up appointments, which may have increased their mortality or SARS-CoV-2 infection rate. This study examined the influence of medication practices on SARS-CoV-2 infection among patients infected with HCV, and whether mortality was associated with SARS-CoV-2 co-infection.

## 2. Materials and Methods

### 2.1. Study Design and Data Collection

We retrospectively analyzed the electronic medical records of 602 patients with first-time positive HCV nucleic acid test results detected at the Chi Mei Medical Center laboratories between 1 May 2021 and 31 March 2023. The sample included patients who visited the hospital for outpatient appointments, health examinations, hospital stays, and emergency care. The patients’ medical records included their basic data, such as age, gender distribution, laboratory data, drug history, COVID-19 vaccination and infection status, and other medical history data. The Taiwan Central Epidemic Command Center’s definition of a confirmed COVID-19 case is as follows: the presence of SARS-CoV-2 in a clinical specimen, a positive SARS-CoV-2 polymerase chain reaction (PCR) test result, or a positive at-home rapid antigen test result as determined by a physician. Patients whose medical records included any of these three criteria were listed as confirmed COVID-19 cases. This study was a non-interventional retrospective study that only gathered the patients’ medical data and did not collect additional samples from the patients.

### 2.2. Ethical Approval

This study was approved by the Institutional Review Board at Chi Mei Medical Center (approval no.: 11111-L02, date of approval: 28 November 2022) and strictly conducted according to the 1964 Declaration of Helsinki.

### 2.3. Laboratory Tests—Nucleic Acid Amplification Testing

We used the Abbott RealTime HCV assay with the Abbott m2000 system. The HCV particles in the sample were broken down to expose the RNA for extraction by magnetic particles. The sample was washed repeatedly to remove other components.

The reverse transcription (RT)-PCR method was used to generate amplified products from the RNA genomes in the clinical HCV specimens. In each PCR cycle, the output of the HCV target sequence was measured using fluorescently labeled oligonucleotide probes in the m2000 system. The probes only generate fluorescent signals when specifically bound to the amplified product. The PCR cycle threshold (Ct) of the fluorescent signal detected by the m2000 system in each sample was plotted onto a standard curve using a calibrator; the sample concentration was the Ct value corresponding to the logarithmic value of the RNA concentration in the original sample. The linearity was 12–100,000,000 IU/mL (1.08–8.00 log IU/mL).

RT-PCR was performed using the Abbott Realtime HCV Genotype II assay (Des Plains, IL, USA) and a PLUS assay (Des Plains, IL, USA) to analyze the HCV subtype in the serum and plasma. The genotype-specific fluorescent probes detect genotypes 1, 2, 3, 4, 5, and 6 and subtypes 1a and 1b.

### 2.4. Data Analysis

In this study, the distributions of continuous variables are reported as the mean and standard deviation (SD), and the distributions of categorical variables are reported by count and percentage. To investigate the SARS-CoV-2 infection-related factors, such as DAA treatment and clinical factors, demographic, COVID-19 vaccination frequency, comorbidities, and infected hepatitis C genotype, a two sample t-test and chi-square test or Fisher’s exact test, if appropriate, were separately applied for continuous and categorical variables. To understand how DAA treatment is related to SARS-CoV-2 infection, univariate and multiple logistic regression were conducted. Odds ratios (ORs) are used to present the measure of association. All statistical analyses and plots were performed using the R software (version 4.2.1). The “networkD3” package in R was also used to generate Sankey plots. All statistical analyses were based on a two-sided hypothesis test with a significance level of *p* < 0.05.

## 3. Results

### 3.1. Baseline Characteristics of the Study Population

This study recruited 602 patients with HCV between May 2021 and March 2023. There were 294 women (48.84%) and 308 men (51.16%). Regarding the age distribution, there were 19 patients aged 20–39 years (3.16%), 192 aged 40–59 years (31.89%), 278 aged 60–79 years (46.18%), and 113 aged ≥80 years (18.77%). Regarding complications, 255 patients had hypertension (43.36%), 154 had diabetes mellitus (25.58%), 117 had cancer (19.44%), 73 had hyperlipidemia (12.13%), 19 had chronic obstructive pulmonary disease (COPD; 3.16%), 58 had CKD (9.63%), and 53 had a cerebrovascular accident (CVA). The direct-acting antivirals (DAAs) used by the patients infected with HCV in this study included Epclusa^®^ (296/462, 64.07%; company: Gilead Sciences Ireland UC; location: IDA Business and Technology Park, Carrigtohill, Co., Cork, Ireland), Harvoni^®^ (1/462, 0.22%; company: Gilead Sciences Ireland UC; location: IDA Business and Technology Park, Carrigtohill, Co., Cork, Ireland), Maviret^®^ (146/462, 31.60%; company: Fournier Laboratories Ireland Limited; location: Anngrove, Carrigtwohill Co., Cork, Ireland), and VOSEVI^®^ (18/462, 3.90%; company: Gilead Sciences Ireland UC; location: IDA Business and Technology Park, Carrigtohill, Co., Cork, Ireland). Regarding HCV genotypes, 27 had genotype 1a (4.49%), 136 had genotype 1b (22.59%), 245 had genotype 2 (40.70%), 15 had genotype 3 (2.49%), 122 had genotype 6 (20.27%), five had a mixed genotype (0.83%), and 25 had no verified genotype (8.64%). There were 35 deaths during the patient recruitment period (5.81%).

### 3.2. Clinical Characteristics of Patients Infected with HCV with Respect to SARS-CoV-2 Infection Status

First, we analyzed the SARS-CoV-2 infection status of the 602 patients infected with HCV (Table 1). The results showed that 59 patients were infected with SARS-CoV-2, while 543 were not. Next, we compared the two groups of patients and found no significant differences in their gender, age, number of vaccinations received, complications, genotype, and mortality. However, we noted that the proportion of patients undergoing DAA treatment was significantly higher (*p* < 0.0001) among those not infected with SARS-CoV-2 (433/543, 79.74%) than among those infected with SARS-CoV-2 (29/59, 49.15%).

### 3.3. Comparison of Patients Undergoing and Not Undergoing DAA Treatment

In order to further elucidate the impact of DAAs on SARS-CoV-2 infection, based on the results in Table 1, we comparatively analyzed the 602 patients infected with HCV with respect to DAA treatment (Table 2). The results showed that the SARS-CoV-2 infection rate was significantly lower (*p* < 0.0001) in patients undergoing DAA treatment (29/462, 6.28%) than those who were not (30/140, 21.43%). The mortality rate was also significantly lower (*p* < 0.0001) among those undergoing DAA treatment (8/462, 1.73%) than among those who were not (27/140, 19.29%).

### 3.4. Analysis of HCV and SARS-CoV-2 Co-Infection with Respect to DAA Treatment and Hepatitis C Genotype

To study how HCV and SARS-CoV-2 co-infection relates to DAA treatment and hepatitis C genotypes, we analyzed the data of 30 patients who did not receive DAA treatment and 29 who did (Table 3). There was a significant difference (*p* < 0.0001) between the DAA treatment and non-DAA treatment groups. Therefore, we further analyzed whether differences existed between different drugs. The results indicated significant differences (odds ratio (OR) = 0.27; 0.24) between the 20 patients who received Epclusa^®^ (68.97%; company: Gilead Sciences Ireland UC; location: IDA Business and Technology Park, Carrigtohill, Co., Cork, Ireland), the nine patients who received Maviret^®^ (30.03%; company: Fournier Laboratories Ireland Limited; location: Anngrove, Carrigtwohill Co., Cork, Ireland), and those who did not receive DAA treatment, suggesting that DAAs generally provide superior protection against SARS-CoV-2, regardless of whether it is Epclusa^®^ (company: Gilead Sciences Ireland UC; location: IDA Business and Technology Park, Carrigtohill, Co., Cork, Ireland) or Maviret^®^ (company: Fournier Laboratories Ireland Limited; location: Anngrove, Carrigtwohill Co., Cork, Ireland) (*p* < 0.05). In the 29 patients with HCV and SARS-CoV-2 co-infection, there was no significant difference in the risk of contracting SARS-CoV-2 between those using Epclusa^®^ (company: Gilead Sciences Ireland UC; location: IDA Business and Technology Park, Carrigtohill, Co., Cork, Ireland) and those using Maviret^®^ (company: Fournier Laboratories Ireland Limited; location: Anngrove, Carrigtwohill Co., Cork, Ireland) (odds ratio (OR) = 0.91, *p* = 0.8131). From the perspective of vaccination frequency, there was no significant difference between patients who received two doses (11.86%) or three doses (52.54%) of the vaccine and those who were unvaccinated. This may be related to the efficacy of the vaccine, including its effectiveness against variant strains and the number of days between vaccination and infection.

**Figure 1:**
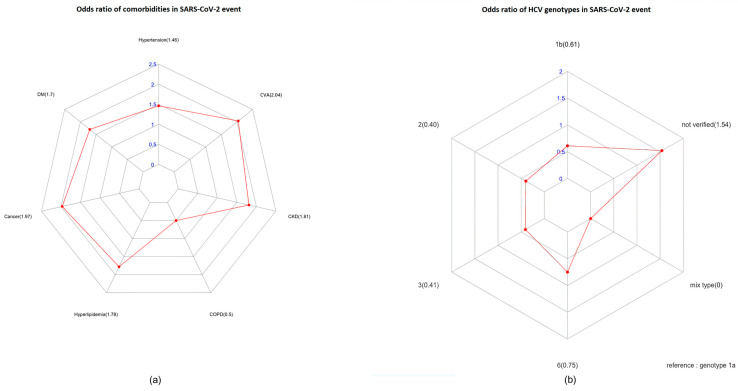
The odds ratio (OR) assay for comorbidities (**a**) and HCV genotypes (**b**) in patients infected with SARS-CoV-2, obtained using R software. (*p* < 0.05).

We statistically examined the presence of complications (hypertension, diabetes, cancer, hyperlipidemia, COPD, CKD, and CVA) in co-infected patients. There was a higher number of patients with hypertension co-infected with SARS-CoV-2 and HCV than in patients solely infected with SARS-CoV-2. Significant differences (*p* = 0.0257) were observed in patients diagnosed with cancer when considering a single complication. Additionally, the odds ratio (OR) analysis demonstrated a higher risk of CVA in co-infected patients (Figure 1a). There were no significant differences between complications and HCV and SARS-CoV-2 co-infection.

Since our hospital is located in a region where HCV genotype 6 is highly prevalent, we analyzed the relationship between genotypes and HCV and SARS-CoV-2 co-infection using a sample of 48 patients with known genotypes. The distribution of genotypes in the sample was follows: four with genotype 1a (6.78%), thirteen with genotype 1b (22.03%), sixteen with genotype 2 (27.12%), one with genotype 3 (1.69%), and fourteen with genotype 6 (23.72%) of the cohort. Additionally, there were eleven HCV-infected patients for whom genotype information was not available (18.64%). The statistical analysis showed no significant relationship between genotype 1a and HCV and SARS-CoV-2 co-infection (*p* > 0.05). Since genotypes 1a, 1b, and 2 are highly prevalent in Taiwan, we found no significant differences between these common genotypes and genotype 6 (*p* > 0.05). Figure 1b shows a significant association between cancer and comorbidities in patients infected with SARS-CoV-2 in the OR analysis (*p* = 0.0366). Therefore, the HCV genotype did not influence the likelihood of concurrent infection with HCV and SARS-CoV-2.

### 3.5. The Disease Severity and Mortality of 29 Patients with SARS-CoV-2 Infection While Undergoing and After Completing DAA Treatment

To retrospectively analyze the relationship between DAA treatment and SARS-CoV-2 infection severity, we analyzed five patients with SARS-CoV-2 infection while undergoing DAA treatment and 24 patients with SARS-CoV-2 infection after completing DAA treatment. The results are shown in Table 4. First, regarding vaccination status, there was no significant difference (*p* = 0.2965) in the number of vaccinations received by the 29 patients during and after DAA treatment. Only five patients had a SARS-CoV-2 infection while undergoing DAA treatment, and 24 patients had a SARS-CoV-2 infection up to 90 days after completing their DAA treatment. Regarding COVID-19 severity and DAA treatment course, 24 patients were diagnosed with mild and moderate-to-severe COVID-19 after DAA treatment, three of whom died (12.5%). All patients diagnosed with COVID-19 during DAA treatment were mild cases, and none died. Therefore, DAA treatment may confer more protection against SARS-CoV-2 infection.

## 4. Discussion

Of the 602 HCV-infected patients, 9.8% (59/602) were infected with SARS-CoV-2. This study found that statistical significance did not exist in terms of patients’ age, gender, vaccination status, and genotype with their SARS-CoV-2 infection status. Regarding comorbidities, the SARS-CoV-2 infection rate was higher in patients diagnosed with hypertension, diabetes, cancer, hyperlipidemia, CKD, and cerebrovascular disease, and the risk of SARS-CoV-2 infection was significantly higher in patients with cancer. Furthermore, 29 patients diagnosed with SARS-CoV-2 were undergoing DAA treatment, 82.8% (24/29) of whom were infected after treatment. Therefore, we surmise that DAA treatment may lower the risk of SARS-CoV-2 infection. Research has shown that hepatitis C increases the hospitalization rate, intensive care unit admission, and in-hospital mortality of patients infected with COVID-19 [15,16,17]. This may be related to HCV’s extrahepatic activity, which strengthens the entry mechanism of SARS-CoV-2 through ACE2 and transmembrane protease serine 2 (TMPRSS2), damaging the endothelial barriers and increasing the expression of inflammatory cytokines [16].

According to one study, individuals with a history of liver disease who contract the novel coronavirus have a 30% increased risk of hospitalization and a threefold higher risk of death compared to those without liver disease. Additionally, individuals with underlying liver cirrhosis have an even higher relative risk [18]. It has been suggested that the replication of hepatitis C virus (HCV) may be inhibited during infection with SARS-CoV-2, but due to a lack of HCV RNA measurements, the complete viral load curve cannot be confirmed [19].

A study conducted in 2021 found that 23.43% of patients with hepatic encephalopathy were infected with COVID-19 [20]. It was observed that individuals who were HCV-positive were more susceptible to SARS-CoV-2 infection in vivo [9]. Among the cases in this study, approximately 9.8% (59 out of 602) of HCV-positive individuals were infected with SARS-CoV-2, possibly suggesting a relationship with the implemented pandemic control measures.

The effectiveness of DAAs in blocking HCV replication has been proven by remarkable advancements in DAA treatment in recent years. The DAAs used by the patients infected with HCV in this study included Epclusa^®^ (company: Gilead Sciences Ireland UC; location: IDA Business and Technology Park, Carrigtohill, Co., Cork, Ireland), Harvoni^®^ (company: Gilead Sciences Ireland UC; location: IDA Business and Technology Park, Carrigtohill, Co., Cork, Ireland), Maviret^®^ (company: Fournier Laboratories Ireland Limited; location: Anngrove, Carrigtwohill Co., Cork, Ireland), and VOSEVI^®^ (company: Gilead Sciences Ireland UC; location: IDA Business and Technology Park, Carrigtohill, Co., Cork, Ireland). Their mechanism of action is to directly act on specific processes in the viral life cycle by interrupting HCV replication at various phases. DAAs target non-structural proteins. They can be divided into three major categories based on their drug mechanism [21,22]:

1. NS3/4A protease inhibitors: The NS3/4A protease is responsible for NS4A, NS4B, NS5A, NS5B from viral polyprotein. Inhibitors of the NS3/4A protease can suppress its activity, thereby effectively inhibiting the replication of the virus. Examples include Glecaprevir (MAVIRET^®^: Fournier Laboratories Ireland Limited/ Anngrove, Carrigtwohill Co., Cork, Ireland) and Voxilaprevir (VOSEVI^®^: Gilead Sciences Ireland UC/IDA Business and Technology Park, Carrigtohill, Co., Cork, Ireland).
2. NS5A inhibitors: These drugs interfere with HCV’s genomic replication and the assembly of its progeny. Examples include VEL (Epclusa^®^: Gilead Sciences Ireland UC; location: IDA Business and Technology Park, Carrigtohill, Co., Cork, Ireland and VOSEVI^®^: Gilead Sciences Ireland UC; location: IDA Business and Technology Park, Carrigtohill, Co., Cork, Ireland), Pibrentasvir (MAVIRET^®^: Fournier Laboratories Ireland Limited/ Anngrove, Carrigtwohill Co., Cork, Ireland), and Ledipasvir (Harvoni^®^: Gilead Sciences Ireland UC/IDA Business and Technology Park, Carrigtohill, Co., Cork, Ireland).
3. NS5B polymerase inhibitors: These drugs inhibit RNA polymerase activity by binding to RNA-dependent RNA polymerase (RdRp) and interrupting virus replication [23]. Examples include SOF (Epclusa^®^: Gilead Sciences Ireland UC; location: IDA Business and Technology Park, Carrigtohill, Co., Cork, Ireland, VOSEVI^®^: Gilead Sciences Ireland UC/IDA Business and Technology Park, Carrigtohill, Co., Cork, Ireland, and Harvoni^®^: Gilead Sciences Ireland UC / IDA Business and Technology Park, Carrigtohill, Co., Cork, Ireland). As a nucleotide prodrug, SOF becomes pharmacologically active through intracellular metabolism and becomes an HCV NS5B RNA polymerase inhibitor. It embeds itself into the HCV RNA through the NS5B polymerase, forcing a chain termination that ends the HCV life cycle [24].

Oral DAAs have a cure rate of 95% and are not restricted by age, liver, and kidney function. This study found that patients infected with HCV who received DAA treatment had lower rates of SARS-CoV-2 infection and mortality (*p* < 0.0001). This finding might be due to the effective elimination of HCV after treatment, reducing the cellular entry of SARS-CoV-2 and the inflammatory responses.

Based on the recommendations that the American Association for the Study of Liver Diseases stated in its guidance document, DAA treatment is recommended for patients newly diagnosed with hepatitis C who are not yet infected with SARS-CoV-2; those who are infected with SARS-CoV-2 can only receive DAA treatment after they are virus-free. Patients infected with SARS-CoV-2 while undergoing DAA treatment should continue treatment but be monitored for drug–drug interactions and hepatic decompensation [25].

Regarding the relationship between the last vaccine dose and SARS-CoV-2 infection, fewer patients were infected with SARS-CoV-2 within 90 days than after 90 days of vaccination. While this finding indicates that vaccination is more effective within three months, the difference was not statistically significant. Given that SARS-CoV-2 has a high mutation rate and different vaccines have varying efficacies, healthcare policymakers should continue to promote COVID-19 vaccination campaigns.

**Figure 2:**
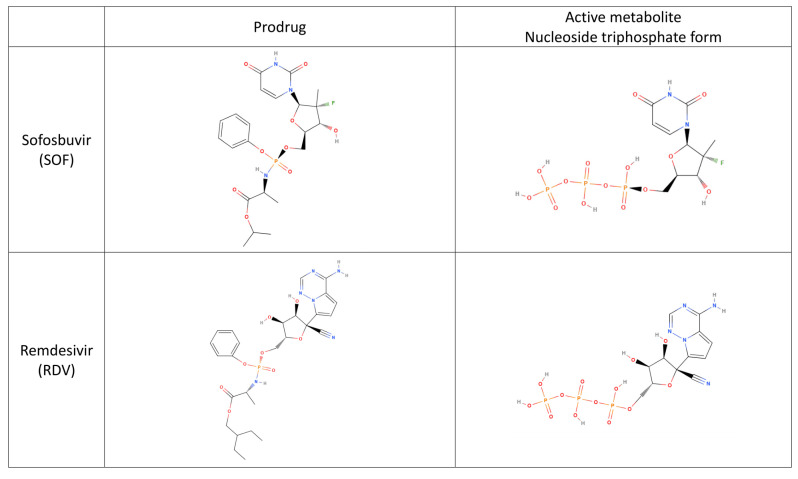
Investigating the chemical structures of SOF and Remdesivir.

Kovalic et al. (2020) highlighted a correlation between chronic hepatitis B or C and SARS-CoV-2 infection severity (pooled OR = 1.48 (95% confidence interval: 1.17–1.87), *p* = 0.001). However, a meta-analysis revealed that SARS-CoV-2 infection does not increase the risk of developing chronic liver disease [26]. Hepatitis C is common in Taiwan, with genotypes 1 and 2 being the most prevalent. The prevalence of hepatitis C genotype 6 is also higher in Southern Taiwan [27]. Among the 97 patients with hepatitis C genotype 6 undergoing DAA treatment in this study, only eight were infected with SARS-CoV-2 (8.24%; 8/97), suggesting no genotype differences concerning SARS-CoV-2 infection. Furthermore, the SARS-CoV-2 infection rate was 9.80% (59/602) among the patients with hepatitis C. Compared to the prevalence rate of SARS-CoV-2, which is over 43.87% in Taiwan, very few patients undergoing DAA treatment for hepatitis C have also been infected with SARS-CoV-2. Significant differences existed between patients taking Epclusa^®^ (company: Gilead Sciences Ireland UC; location: IDA Business and Technology Park, Carrigtohill, Co., Cork, Ireland) and Maviret^®^ (company: Fournier Laboratories Ireland Limited; location: Anngrove, Carrigtwohill Co., Cork, Ireland) and those not undergoing DAA treatment. SOF in Epclusa^®^ (company: Gilead Sciences Ireland UC; location: IDA Business and Technology Park, Carrigtohill, Co., Cork, Ireland) binds to the SARS-CoV-2 RdRp, inhibiting the virus’ polymerase binding and exonuclease-based proofreading activity [28,29]. The structure of SOF is shown in Figure 2. In addition to SOF, antivirals such as galidesivir, tenofovir, and ribavirin can also inhibit SARS-CoV-2 replication by binding to RdRp [30]. However, in this study, five patients were diagnosed with mild COVID-19 while undergoing DAA treatment. While clinical studies have found that hepatitis C medications and COVID-19 antivirals share similar mechanisms of action and constituents, both are still distinct drug types. Patients living with chronic hepatitis C, cirrhosis, hepatic resection, a removed gallbladder, or diabetes might have a higher risk of SARS-CoV-2 infection due to their weakened immune system [31,32].

In this study, five patients were diagnosed with COVID-19 while undergoing DAA treatment for hepatitis C. The patients had to complete follow-up visits and monitor their blood parameters during therapy. The risk of SARS-CoV-2 infection was higher for these patients, presumably because they had to make return visits to the hospital. One study found that hospital workers faced a higher risk of SARS-CoV-2 infection than the general public [33]. Frontline healthcare workers also had a higher infection risk than those who did not have direct contact with patients [34]. This exposure risk decreased as the vaccination rate increased. Furthermore, there were no deaths among the patients infected with SARS-CoV-2 while undergoing DAA treatment.

Based on their medical history, all 36 patients who died had a history of chronic diseases, with cirrhosis and liver cancer each accounting for 45%, while diabetes accounted for 22%. Most deaths were caused by sepsis (15/36), liver cancer (10/36), organ failure (heart, liver, or lungs), and hemorrhagic shock. Most deaths were associated with patients’ pre-existing conditions, while two died from COVID-19. Therefore, the general public should monitor their health, properly manage their chronic conditions, and seek timely treatment during the COVID-19 pandemic.

When the COVID-19 pandemic began, the CDC announced that individuals with positive rapid antigen test results were confirmed cases. However, this approach does not reflect the SARS-CoV-2 viral load or genotype, and this study did not compare most patients’ liver function or other examination parameters, which is one limitation of this study. By tracing the SARS-CoV-2 infection status of patients infected with HCV, we found that the co-infection rate of HCV and SARS-CoV-2 was not high. In addition to vaccination policies, quarantining patients with positive rapid antigen test results at home minimized their risk of exposure and reduced their co-infection rate.

## 5. Conclusions

**Figure 3:**
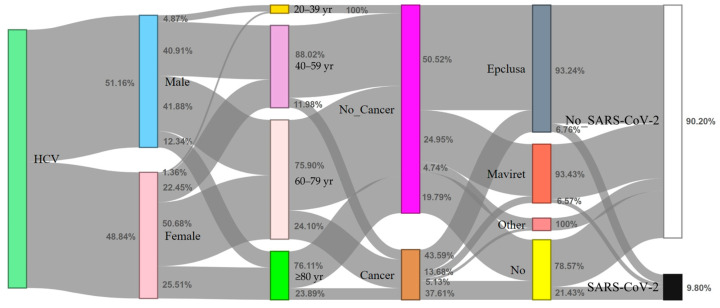
Sankey diagram depicting the distribution of HCV-infected individuals in relation to SARS-CoV-2 infection status (*n* = 602). The content of the figure illustrates the distribution of HCV-infected individuals concerning four variables (gender, age, cancer, and DAA medication) in relation to their SARS-CoV-2 infection status using a Sankey diagram. The description also mentions the visual elements used in the diagram, such as vertical colored bars and gray flow arrows.

SARS-CoV-2 infection triggered a global pandemic. According to our findings, HCV-infected patients receiving DAA treatment exhibited lower rates of SARS-CoV-2 infection and mortality compared to those not undergoing DAA therapy (Figure 3). These observations contribute to the understanding of the interplay between HCV, DAA treatment, and SARS-CoV-2 infection. However, further research is necessary to comprehensively elucidate potential mechanisms and outcomes. This information is crucial for shaping healthcare strategies. 

## Figures and Tables

**Table 1:** Comparison between group not infected with SARS-CoV-2 and group infected with SARS-CoV-2 (*n* = 602).

Characteristics, *n* (%)	Not Infected with SARS-CoV-2 *n* = 543	SARS-CoV-2 Infection *n* = 59	*p*-Value
Gender			0.5257
Female	268 (49.36)	26 (44.07)	
Male	275 (50.64)	33 (55.93)	
Age, mean (SD)	64.24 (14.11)	67.68 (14.40)	0.0763
Age group			0.5158
20–39 year	18 (3.31)	1 (1.69)	
40–59 year	177 (32.60)	15 (25.42)	
60–79 year	249 (45.86)	29 (49.15)	
≥80 year	99 (18.23)	14 (23.73)	
Vaccinate			0.479
0	129 (23.76)	19 (32.20)	
1	33 (6.08)	2 (3.39)	
2	74 (13.63)	7 (11.86)	
≥3	307 (56.54)	31 (52.54)	
Comorbidities (*n* = 389)			
Hypertension	225 (41.44)	30 (50.85)	0.2111
Diabetes mellitus	133 (24.49)	21 (35.59)	0.0894
Cancer	99 (18.23)	18 (30.51)	0.0366
Hyperlipidemia	62 (11.42)	11 (18.64)	0.1601
COPD	18 (3.31)	1 (1.69)	0.7765
CKD	49 (9.02)	9 (15.25)	0.1909
CVA	44 (8.10)	9 (15.25)	0.1098
Genotype			0.0532
1a	23 (4.24)	4 (6.78)	
1b	123 (22.65)	13 (22.03)	
2	229 (42.17)	16 (27.12)	
3	14 (2.58)	1 (1.69)	
6	108 (19.89)	14 (23.73)	
mix type	5 (0.92)	0 (0)	
not verified	41 (7.55)	11 (18.64)	
DAA treatment	433 (79.74)	29 (49.15)	<0.0001
Mortality	28 (5.16)	7 (11.86)	0.0721

**Table 2:** Comparison between non-DAA treatment and DAA treatment groups (*n* = 602).

Characteristics, *n* (%)	Non-DAA Treatment *n* = 140	DAA Treatment *n* = 462	*p*-Value
Gender			0.9803
Female	69 (49.29)	225 (48.70)	
Male	71 (50.71)	237 (51.30)	
Age, mean (SD)	70.76 (15.41)	62.70 (13.22)	
SARS-CoV-2 infection	30 (21.43)	29 (6.28)	<0.0001
Mortality	27 (19.29)	8 (1.73)	<0.0001

**Table 3:** Analysis of SARS-CoV-2 infection-related factors in patients infected with HCV (*n* = 59).

Item	Event of Infected with SARS-CoV-2, *n* (%)	OR (95%CI)	*p*-Value	Adjusted OR (95%CI)	*p*-Value	Adjusted OR (95%CI)	*p*-Value
DAA treatment							
No	30 (50.85)	ref					
Yes	29 (49.15)	0.25 (0.14–0.43)	<0.0001	0.27 (0.15–0.49)	<0.0001		
DAA treatment							
Non-DAA treatment	30 (50.85)	ref					
Epclusa	20 (68.97)	0.27 (0.14–0.48)	<0.0001			0.29 (0.15–0.54)	0.0001
Maviret	9 (30.03)	0.24 (0.1–0.51)	0.0004			0.28 (0.11–0.62)	0.0025
Vosevi	0 (0)	-	-			-	-
others	0 (0)	-	-			-	-
Vaccinate							
0	19 (32.20)	ref					
1	2 (3.39)	0.41 (0.06–1.52)	0.2479				
2	7 (11.86)	0.64 (0.24–1.54)	0.3416				
≥3	31 (52.54)	0.69 (0.38–1.28)	0.2229				
Comorbidities							
Hypertension	30 (11.76)	1.46 (0.85–2.51)	0.1666				
DM	21 (13.64)	1.7 (0.95–2.98)	0.0659				
Cancer	18 (15.38)	1.97 (1.06–3.52)	0.0257	1.46 (0.76–2.70)	0.2361	1.47 (0.77–2.72)	0.2314
Hyperlipidemia	11 (15.07)	1.78 (0.84–3.49)	0.1104				
COPD	1 (5.26)	0.5 (0.03–2.5)	0.5073				
CKD	9 (15.52)	1.81 (0.79–3.76)	0.1283				
CVA	9 (16.98)	2.04 (0.89–4.25)	0.0707				
Genotype							
1a	4 (6.78)	ref					
1b	13 (22.03)	0.61 (0.19–2.3)	0.4182				
2	16 (27.12)	0.4 (0.13–1.49)	0.1287				
3	1 (1.69)	0.41 (0.02–3.14)	0.4463				
6	14 (23.72)	0.75 (0.24–2.81)	0.6309				
mix type	0 (0)	-	-				
not verified	11 (18.64)	1.54 (0.47–6.07)	0.4977				
Genotype_2							
1a1b2	33 (55.93)	ref					
3	1 (1.69)	0.81 (0.04–4.24)	0.8426				
6	14 (23.72)	1.47 (0.74–2.8)	0.2506				
mix type	0 (0)	-	-				
not verified	11 (18.64)	3.05 (1.38–6.34)	0.0038				

**Table 4:** HCV DAA treatment and patients infected with SARS-CoV-2 (*n* = 29).

Characteristics, *n* (%)	DAA Within Treatment *n* = 5	Complete DAA Treatment *n* = 24	*p*-Value/ Nonparametric Fisher/MWU Test
Vaccinate			0.2965
0	0 (0)	7 (29.17)	
≥1	5 (100)	17 (70.83)	
SARS-CoV-2 infection severity			0.9999
Mild	5 (100.00)	21 (87.50)	
Moderate, Severe	0 (0)	3 (12.50)	
Mortality	0 (0)	3 (12.50)	

## Data Availability

All data are fully available without any restriction upon reasonable request.
